# Integrin beta1 (ITGB1) as a prognostic marker in esophageal adenocarcinoma

**DOI:** 10.1038/s41598-022-25071-y

**Published:** 2022-12-01

**Authors:** Alexander I. Damanakis, Isabell Wahler, Hans Fuchs, Heike Löser, Wolfgang Schröder, Thomas Zander, Seung-Hun Chon, Christiane Bruns, Alexander Quaas, Florian Gebauer

**Affiliations:** 1 Department of General, Visceral and Cancer Surgery, University Hospital of Cologne, Kerpener Strasse 62, 50937 Cologne, Germany; 2 Institute of Pathology, University Hospital of Cologne, Cologne, Germany; 3 Department I of Internal Medicine, Center for Integrated Oncology Aachen Bonn Cologne Duesseldorf, Gastrointestinal Cancer Group Cologne GCGC, University Hospital of Cologne, Cologne, Germany

**Keywords:** Biomarkers, Medical research, Gastroenterology, Gastrointestinal cancer, Oesophageal cancer

## Abstract

Today, individual prognosis in patients with adenocarcinoma of the esophagus (EAC) is based on post-surgical TNM staging and valid biomarkers are still not implemented. Integrin beta1 (ITGB1) is widely expressed in epithelial cells and promotes cell adhesion and growth. Its impact on tumor progression was described for different tumor entities before, data on its function as a potential biomarker in EAC is not available. Aim of the study is to evaluate the expression level of ITGB1 in a large collective of EAC and its impact on patients´ prognosis. 640 patients with esophageal adenocarcinoma were analyzed immunohistochemically for ITGB1. The data was correlated with long term outcome, clinical, pathological and molecular data (TP53, HER2/neu, c-myc, GATA6, PIK3CA and KRAS). Of 640 patients to be analyzed, 127 (19.8%) showed expression of ITGB1. ITGB1 expression was associated with lymph node metastasis, expression of integrin alphaV and KRAS mutation status. Patients with high ITGB1 expression showed impaired overall survival (22.5 months (95% CI 15.3–29.7 months), vs. 34.1 months (95% CI 25.3–42.4 months), P = 0.024). This effect was particularly evident in the group of patients undergoing primary surgery without prior neoadjuvant therapy (10.2 months (95% CI 1.9–41.7 months) vs. 31.4 months (95% CI 21.1–144.2 months, P = 0.008). ITGB1 was also an independent prognostic marker in multivariable analysis (HR 1.696 (95% CI 1.084–2.653, P = 0.021) in patients that underwent primary surgery. We demonstrate for the first time the prognostic significance of ITGB1 expression in a large EAC patient population.

## Introduction

Esophageal cancer ranks seventh in terms of incidence and sixth in mortality worldwide^1^. A rising incidence of esophageal adenocarcinoma (EAC) is described in high-income western countries and is expected to rise even further^2^. Establishing multimodal treatment protocols shows promising results in the treatment of EAC. However, overall-survival of patients with EAC remains limited^3,4^ Excluding clinical parameters like age and histopathological grading there are barely any prognostic factors established for risk stratification in patients with EAC. To individualize the current treatment protocols the use of prognostic biomarkers should be implied to identify patients benefiting from multimodal therapy including chemotherapy, radiotherapy, surgery and immunotherapy^5,6^.

Integrins are heterodimeric transmembrane proteins which regulate cell–cell and cell-extracellular matrix (ECM) interactions^7^. There are 24 known Integrins which are structurally made from a combination of a beta and an alpha subunit^8^. Today 18 alpha and 8 beta integrin subunits are described. Integrins play a major part in organizing cytoskeleton, activating intracellular signal pathways thereby promoting cell survival as well as mediating cell responses to growth factors and cytokines^8,9^. Due to these functions it is that an increasing interest has risen in the role integrins play in malignant diseases. Several integrins have been found to be involved in tumorigenesis, tumor progression and in the metastatic cascade for different tumor entities. Our group was able to show the impact of integrin alpha V expression on patients’ prognosis in esophageal cancer^10^.

In the present study, we are focusing on Integrin beta1 (ITGB1), which has been described as tumor progressor in various tumor entities like lung cancer, colon cancer^11^ and prostate cancer^12^.

To date, there is no analysis of ITGB1 expression in esophageal adenocarcinoma. The aim of this study was to analyze the expression of ITGB1 in esophageal adenocarcinoma and possibly correlate the expression profile with clinico-pathological, molecular and survival data.

## Patients and Methods

### Patients and tumor samples

Formalin-fixed and paraffin embedded tumor tissue of 685 patients with esophageal adenocarcinomas that underwent primary surgical resection or resection after neoadjuvant therapy between 1999 and 2014 at the Department of General, Visceral and Cancer Surgery, University of Cologne, Germany was analyzed as previously described^10,13,14^. The standard surgical procedure consisted of a transthoracic en-bloc esophagectomy with two-field lymphadenectomy (abdominal and mediastinal lymph nodes), reconstruction by formation of a gastric tube with intrathoracic esophagogastrostomy (Ivor-Lewis esophagectomy)^15^. The abdominal phase was predominantly performed as a laparoscopic procedure (hybrid Ivor-Lewis esophagectomy). Technical details of this operation are described elsewhere^16–18^. Patients with locally advanced esophageal cancer (cT3) or evidence for loco regional lymph node metastasis in clinical staging received preoperative chemoradiation (5-Fluouracil, cisplatin, 40 Gy) or chemotherapy alone. Follow-up data were available for all patients (Table 1).

**Table 1:** Clinico-pathological data of the entire patient cohort.

	Total		Integrin beta1 expression				P value
			Negative		Positive		
	N	%	N	%	N	%	
	640	100	513	80.2	127	19.8	
**Sex**							
Female	76	11.9	59	77.6	17	22.4	0.542
Male	564	88.1	454	80.5	110	19.5	
**Age group**							
< 65 years	327	53.0	264	80.7	63	19.3	0.762
> 65 years	290	47.0	231	79.7	59	20.3	
**Neoadjvuant therapy**							
No	265	41.4	213	80.4	52	19.6	0.494
Yes	375	58.6	300	80.0	75	20.0	
**Tumor stage**							
(y) pT1	133	20.9	112	84.2	21	15.8	0.103
(y) pT2	123	19.4	105	85.4	18	14.6	
(y) pT3	359	56.5	276	76.9	83	23.1	
(y) pT4	20	3.1	15	75.0	5	25.0	
**Lymph node stage**							
(y) pN0	259	40.6	222	85.7	37	14.3	0.039
(y) pN1	210	32.9	162	77.1	48	22.9	
(y) pN2	88	13.8	67	76.1	21	23.9	
(y) pN3	81	12.7	61	75.3	20	24.7	
**UICC stage**							
I	101	15.9	86	85.1	15	14.9	0.208
II	80	12.6	67	83.8	13	16.3	
III	287	45.2	229	79.8	58	20.2	
IV	167	26.3	126	75.4	41	24.6	
***Kras* mutation**							
No	415	82.0	352	84.8	63	15.2	0.044
Yes	91	18.0	69	75.8	22	24.3	

Single spot tissue micro arrays (TMA) were built for immunohistochemical analyses. TMA construction was performed as previously described^19,20^. In brief, tissue cylinders with a diameter of 1.2 mm each were punched from selected tumor tissue blocks using a self-constructed semi-automated precision instrument and embedded in empty recipient paraffin blocks. 4 μm sections of the resulting TMA blocks were transferred to an adhesive coated slide system (Instrumedics Inc., Hackensack, NJ) for immunohistochemistry. All procedures performed in studies involving human participants were in accordance with the ethical standards of the institutional research committee and with the 1964 Helsinki declaration and its later amendments or comparable ethical standards. The present study was ethically approved by the University of Cologne Ethics Committee (Reference No. 13-091) and written informed consent was obtained from all patients.

### Immunohistochemistry for Integrin beta1 (ITGB1)

Immunohistochemistry (IHC) was performed on TMA slides using the Integrin beta1 rabbit monoclonal antibody (A-4; dilution 1:100; Santa Cruz, USA). Staining and scoring procedures were conducted as previously described^20–23^. All immunohistochemical stainings were performed using the Leica BOND-MAX stainer (Leica Biosystems, Germany) according to the protocol of the manufacturer.

**Figure 1:**
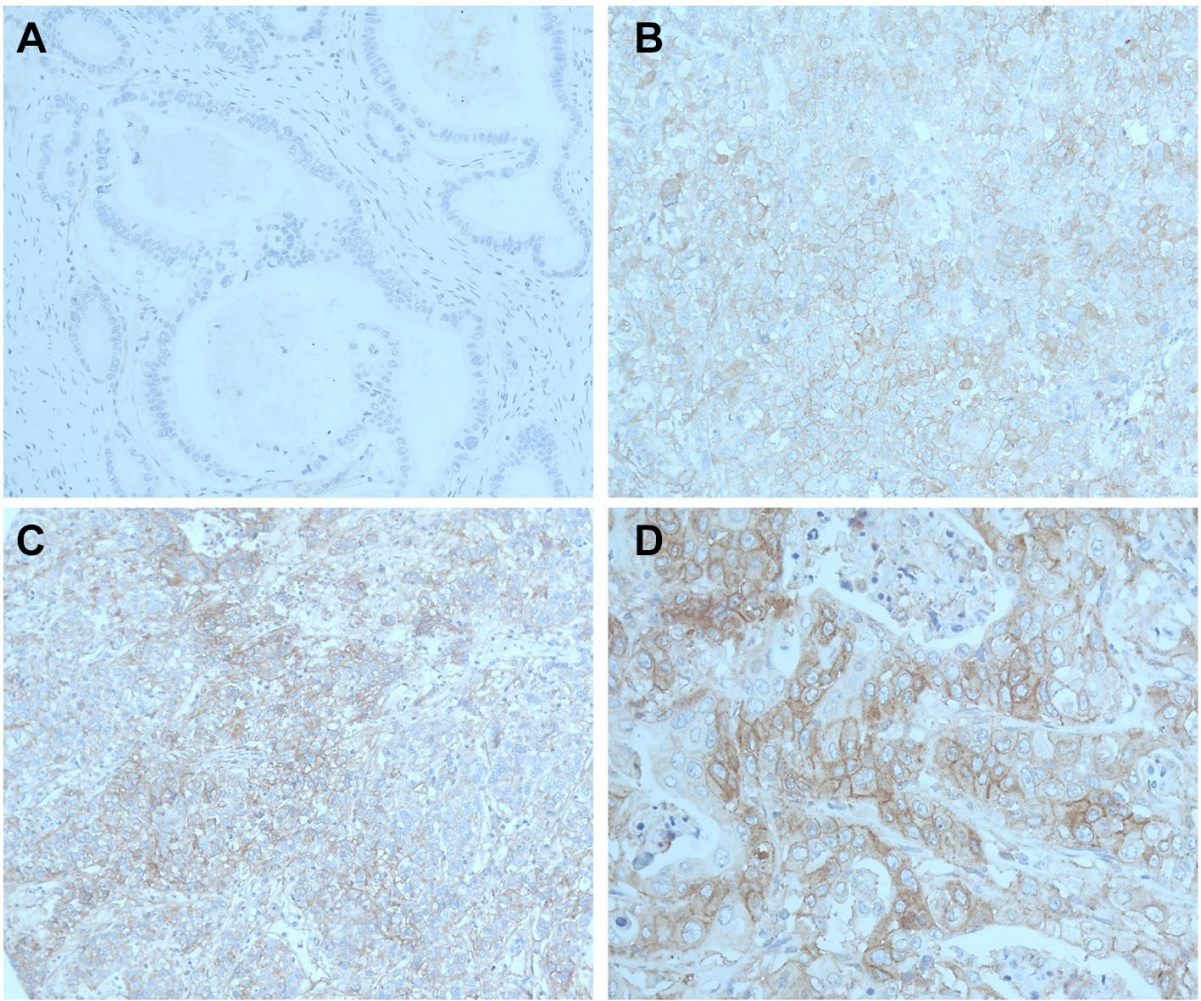
Immunohistochemistry staining for integrin beta1 (ITGB1). Negative tumor sample for ITGB1 (**A**), weak (**B**), moderate (**C**) and strong staining intensity (**D**).

The membraneous staining pattern was scored manually and independently by two pathologists (A.Q. and H.L.) according to a 4-tier-scoring system (Fig. 1). Score 3 + was defined as a strong staining of ≥ 30% of tumor cells or moderate staining ≥ 70%. A weak staining in > 70% or moderate staining in > 30 and ≤ 70%, or a strong staining in ≤ 30% of tumor cells was considered as Score 2 + . Score 1 + was assigned when ≤ 70% of tumor cells were weakly positive or ≤ 30% were moderately stained. Less staining was defined as negative (Score 0). Discrepant results were resolved by consensus review.

Expression of ITGB1 was correlated with molecular markers including analysis of TP53, Her2/neu, c-myc, GATA6, PIK3CA mutations and KRAS amplification.

### Statistical analysis

Clinical data were collected prospectively and analyzed according to a standardized protocol as previously described^13,14,20^. SPSS Statistics for Mac (Version 21, SPSS) was used for statistical analysis. Interdependence between staining results and clinical data were calculated using the chi-squared and Fisher’s exact tests, and displayed by cross-tables. Survival curves were plotted using the Kaplan–Meier method and analyzed using the log-rank test. All tests were two-sided. P values < 0.05 were considered statistically significant.

## Results

### Patients’ baseline characteristics

On the TMA a total of 640 patients of 685 (93.2%) were immunohistochemically interpretable for ITGB1. Reasons for the non-informative cases were missing tissue samples or the absence of distinct cancer tissue in the TMA spot. Patients were predominantly males (n = 564, 88.1%), females n = 76, 11.9%. The median age of the entire patient cohort at the time of diagnosis was 65.2 years (range 33.6–85.6 years). In 333 patients (56.0%) a neoadjuvant treatment (chemo- or radiochemotherapy) was performed before surgery.

### Expression of ITGB1 in esophageal adenocarcinoma

**Figure 2:**
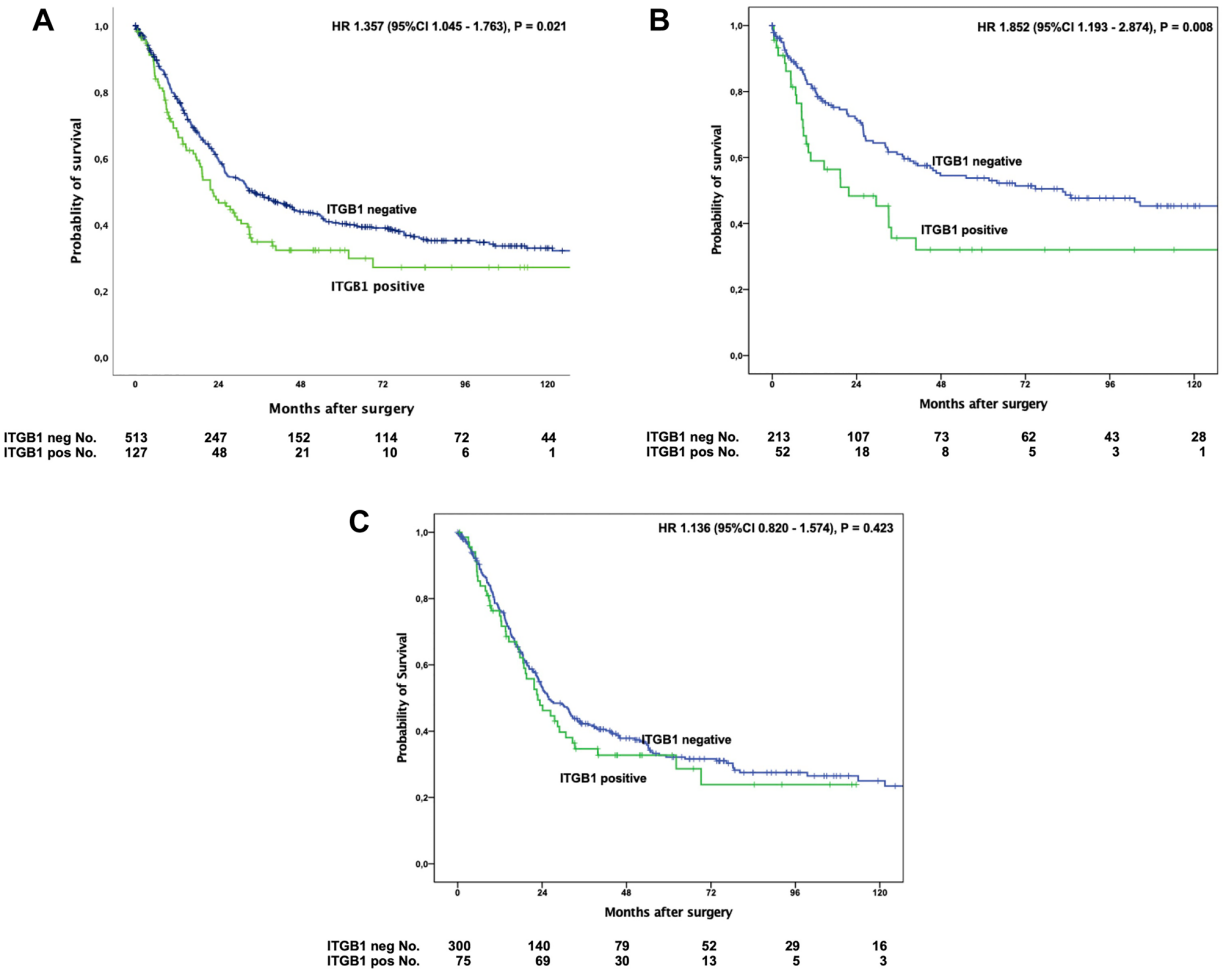
Kaplan–Meier survival analysis for overall survival of the entire patient cohort Kaplan–Meier survival analysis for overall survival of the entire patient cohort (**A**). Median OS in patients with presence of ITGB1 was 22.5 months (95% confidence interval (95% CI) 15.3–29.7 months), and 34.1 months (95% CI 25.3–42.4 months, P = 0.024) without ITGB1 expression. Median OS in patients after primary surgery (**B**) with ITGB1 expression in the tumor was 10.2 months (95% CI 1.9–41.7 months) compared to 31.4 months (95% CI 21.1–144.2 months, P = 0.008) in the group without ITGB1 expression. Patients that received neoadjuvant treatment before surgery (**C**) showed a median OS with ITGB1 expression of 22.7 months (95%CI 15.3–30.2 months) vs. 25.9 months without ITGB1 expression (95% CI 20.6–31.2 months), P = 0.423).

Expression of ITGB1 was detected in 127 patients (19.9%) (Fig. 2). ITGB1 expression was associated with presence of lymph node metastasis (P = 0.039) and Integrin alpha V (ITGAV) expression (P < 0.001). A correlation of ITGB1 expression with molecular marker could only be seen for *KRAS* mutation status (P = 0.044) (Table 1).

### ITGB1 expression marks poor outcome in patients without neoadjuvant treatment

Patients with expression of ITGB1 (score 1 +, 2 +, 3 +) in the primary tumor showed an impaired overall survival compared to patients without ITGB1 expression (score 0). Median OS in patients with presence of ITGB1 was 22.5 months (95% confidence interval (95% CI) 15.3–29.7 months), median OS in patients without ITGB1 expression was 34.1 months (95% CI 25.3–42.4 months, P = 0.024). Stratification in patients with and without any kind of neoadjuvant treatment revealed that the survival difference is mainly driven by the group of patients that underwent primary surgery without neoadjuvant treatment. In this group, patients with ITGB1 expression in the tumor showed a median OS of 10.2 months (95% CI 1.9–41.7 months) compared to a median OS of 31.4 months (95% CI 21.1–144.2 months, P = 0.008) in the group without ITGB1 expression (Fig. 2).

In the patient cohort after neoadjuvant treatment, an ITGB1 associated survival difference could not be observed. The median overall survival did not differ significantly between the two groups (median OS in patients with ITGB1 expression 22.7 months (95% CI 15.3–30.2 months) vs. 25.9 months without ITGB1 expression (95% CI 20.6–31.2 months), P = 0.423).

A multivariate cox-regression model showed that ITGB1 is an independent prognostic factor in the group of patients without neoadjuvant treatment (hazard ratio (HR) 1.171 (95% CI 1.089–2.707), P = 0.020) but failed to serve as prognostic marker in the patients group after neoadjuvant treatment (HR 1.121 (95% CI 0.806–1.557), P = 0.498) (Tables 2 and 3).

**Table 2:** Multivariate cox-regression analysis for patients after primary surgery.

	Hazard ratio	95% Confidence interval		
		Lower	Upper	P value
Sex (male vs. female)	1.108	0.582	2.109	0.754
Age group (< 65 years vs. > 65 years)	1.387	0.921	2.090	0.117
Tumor stage (pT1/2 vs. pT3/4)	2.355	1.528	3.627	< 0.001
Lymph node metastasis (pN0 vs. pN +)	2.972	1.885	4.686	< 0.001
Integrin beta1 expression (negative vs. positive)	1.696	1.084	2.653	0.021

**Table 3:** Multivariate cox-regression analysis for patients after neoadjuvant (chemo) radiotherapy.

	Hazard ratio	95% Confidence interval		
		Lower	Upper	P value
Sex (male vs. female)	1.324	0.780	2.013	0.190
Age group (< 65 years vs. > 65 years)	1.105	0.847	1.440	0.462
Tumor stage (pT1/2 vs. pT3/4)	0.912	0.684	1.215	0.530
Lymph node metastasis (pN0 vs. pN +)	2.234	1.659	3.007	< 0.001
Integrin beta1 expression (negative vs. positive)	1.121	0.806	1.557	0.498

## Discussion

To date, the impact of ITGB1 on survival in esophageal adenocarcinoma has not been studied. In our analysis, we were able to examine 640 primary tumors regarding ITGB1 expression. Approximately 20% of the tumors were positive for ITGB1 and were associated with a significantly worse prognosis than ITGB1 negative tumors. This effect is stronger in the group of patients without neoadjuvant therapy than in the group of patients who received (radio) chemotherapy prior to esophagectomy.

The role of integrins in tumor progression has received more and more attention in recent years. In the past, integrins were primarily described as interaction partners of epithelial cells to the extracellular matrix, but recent data show a direct influence on tumor progression of different integrin subtypes in a variety of tumor entities^7,8^. In our preliminary work, we have already demonstrated that expression of integrin alpha V has an impact on overall survival of patients with adenocarcinoma of the esophagus^10^. In the present study, we focused on the most common beta subunit of integrines. Integrin beta 1 is the most physiologically abundant beta subunit and together with a variety of alpha subunits forms a multitude of heterodimer combinations. The physiologically mediated functions in humans are diverse: for example, ITGB1 forms a so-called RGD (Arg-Gly-Asp) receptor binding domain in the combination of alpha5 and alpha8. The combination alpha4/beta1 enables specific leukocyte binding as well as binding to laminin and various collagens^24,25^. The role of ITGB1 in tumor progression has been demonstrated for several tumor entities, including lung, prostate, breast, and colorectal cancer^26^.

The mechanisms described via which ITGB1 induces tumor progression are diverse. In the past, the focus was on the analysis of an altered extracellular matrix within the tumor and thus a modified signal transduction after bidding of ITGB1 expressing tumor cells to the ECM^26^.

In addition to the direct interaction of integrins with the ECM, the influence of TGFbeta signaling in dependence of integrin expression on the remodeling of tumor microenvironment could be shown^27^. In this context, TGF beta mediates remodeling of the ECM which mediates tumor progression. In addition to local effects of integrin expression on tumor cells, detection of ITGB1 in tumor exosomes was shown to provide a premetastatic niche for lung metastases in pancreatic cancer. The effect was demonstrated via gene upregulation of S100 in lung fibroblasts which subsequently promoted the formation of pulmonary metastases^28^.

In prostate cancer, interactions between the transmembrane molecule Trop-2 and beta1 integrins results in re-localization of integrin beta 1 at the leading edges and can promote prostate cancer cell migration on fibronectin^12^. Metastatic and migratory capabilities of prostate cancer cells are in part integrin beta 1 dependent and rely on the Trop-2 promotion^29^. Trop-2 has recently gained attention as a clinical study in patients with triple-negative breast cancer treated with the drug Savituzumab govitecan (SG), a combination of anti-Trop-2 antibody and SN-38 (active metabolite of Irinotecan) could show significant positive effects on progression free and overall survival^30^. In a phase I/II study for SG’s use in metastatic epithelial cancers, 19 esophageal cancer patients were included of which 10 (52.6%) showed stable disease for at least 3.4 months^31^. The link between ITGB1 and Trop-2 mediated tumor progression could be of interest for the future as ITGB1 expression may be a potential biomarker of response prediction for SG therapy. However, future research should study the role of Trop-2 and its interactions with integrin beta 1 in EAC.

There are no data concerning the effect of neoadjuvant therapy on protein expression of ITBG1 in esophageal cancer. In our data, we find more pronounced effects on overall survival in the group of patients who did not receive preoperative (radio)-chemotherapy. One hypothesis is that neoadjuvant therapy leads to a variety of epigenetic changes within the tumor and thus integrin mediated effects on natural tumor progression are no longer detectable. We could detect similar effects for a variety of biomarkers in our collective^13,21–23^. For example, we demonstrated this for integrin alphaV, dickkopf-2, VISTA, and other biomarkers where prognostic relevance was not present after administration of neoadjuvant therapy.

Findings in other studies had proposed that up-regulation of ITGB1 would contribute to cell survival after radiation exposure in various cancers, thus facilitating resistance^32^. Our collective includes solely patients with esophageal adenocarcinoma. A recent publication by Xie and colleagues revealed very similar results in esophageal squamous cell carcinoma (ESCC) in 278 patients^33^. Only 6 (2.1%) patients had received any form of pretreatment in their ESCC cohort. They report high expression of ITGB1 in 179 patients (64%), whereas ITGB1 positivity in our cohort of patients after primary surgery was 19.6%. Their findings regarding the effect on survival are in line with our findings, considering the pronounced effects in our primary surgery cohort, and the fact that their collective almost entirely consists of patients that underwent primary surgery. This finding is even more interesting, as genomic data reveals that esophageal squamous carcinoma and esophageal adenocarcinoma can likely be considered two different diseases^34^. In our collective, ITGB1 positivity was not associated with a difference in survival after neoadjuvant therapy. It could be hypothesized that neoadjuvant therapies’ positive anti-tumor effect levelled the survival disadvantage of the ITGB1 positive patients compared to the ITGB1 negative patients. The median OS between the ITGB1 positive group after neoadjuvant therapy was 22.7 months and the untreated ITGB1 positive group had a 10.2 month median OS. Considering that patients who receive neoadjuvant therapy presented with a more progressed clinical tumor stage to receive pre-treatment in the first place, the survival difference in these two groups is notable. Especially, as no correlation of ITGB1 with the UICC stage was observed.

In conclusion, our findings support integrin beta1 as a possible prognostic biomarker in esophageal adenocarcinoma. The negative effect on survival is particularly evident in the group of primary resected patients in our cohort. The extent to which individual patient prognosis can be predicted in pretherapeutic biopsies is currently under investigation and could potentially influence treatment decisions for and against neoadjuvant therapy in the future. Association of integrin beta expression with other targetable molecules such as Trop-2 should be the object of further studies in EAC.

## Data Availability

Data available on request to the corresponding author due to privacy/ethical restrictions.
